# Comment on Ashraf et al. Worsening situation of smog in Pakistan: A tale of three cities. *Ann Med Surg (Lond)* 2022, 79, 103947

**DOI:** 10.1016/j.amsu.2022.104966

**Published:** 2022-11-19

**Authors:** Muhammad Bilal, Gerrit de Leeuw, Janet E. Nichol, Max P. Bleiweiss, Leiku Yang, Huabin Chai, Alaa Mhawish, Md Arfan Ali

**Affiliations:** School of Surveying and Land Information Engineering, Henan Polytechnic University, Jiaozuo, China; Royal Netherlands Meteorological Institute (KNMI), R & D Satellite Observations, De Bilt, Netherlands; Aerospace Information Research Institute, Chinese Academy of Sciences (AirCAS), Beijing, China; Department of Geography, School of Global Studies, University of Sussex, Brighton, United Kingdom; Department of Entomology, Plant Pathology, and Weed Science, New Mexico StateUniversity, Las Cruces, NM, United States; School of Surveying and Land Information Engineering, Henan Polytechnic University, Jiaozuo, China; School of Marine Sciences, Nanjing University of Information Science and Technology, Nanjing, China

Recently, a short communication entitled “*Worsening situation of smog in Pakistan: A tale of three cities”* by Ashraf et al. (2022), published in *Annals of Medicine and Surgery*, discussed the rapid rise in smog and air pollution in Pakistani cities including Lahore, Gujranwala, and Faisalabad, in spite of the implementation of some policy measures by the Punjab government to reduce emission. In short comment, we do not argue about the conclusion that more stringent measures are needed. However, we do take issue with the presentation of the data, in particular the interpretation of the air quality index (AQI). For example:

1. Misinterpretation of AQI: The authors stated that (a) *According to recent data, the Air Quality Index (AQI) in certain areas of Lahore has frequently exceeded* 400 μg/m^3^ *in the month of November 2021 while the safe AQI value is considered below* 50 μg/m^3^ *(their reference 1); (b) According to recent IQAir reports (November 2021), air quality data of Faisalabad, Lahore and Gujranwala is 297.*2 μg/m^3^*, 271.*8 μg/m^3^ *and 201.*6 μg/m^3^ *respectively; and (c) Figure 1.*

The AQI discussed in this paper is provided by IQAir (https://www.iqair.com/) which is based on near-surface measurements of the mass concentrations of particulate matter PM_2.5_ (particles with diameters smaller than 2.5 μm) and PM_10_ (smaller than 10 μm) weighted after bringing the particles in low relative humidity environment to remove water, and concentrations of four trace gases, i.e., SO_2_, NO_2_, CO, and O_3_. The AQI for each of these species is determined as explained in, e.g., Fan et al. [1] following Yuan and Yang [2] and according to the “Technical Assistance Document for the reporting of Daily Air Quality – the Air Quality Index (AQI) by the EPA (U.S. Environmental Protection Agency) published in September 2018. The equation used in these publications clearly shows that the AQI is dimensionless, and the hourly (daily) value is determined by the species with the highest AQI during that hour (day). On their website (https://www.iqair.com/newsroom/what-is-aqi), IQAir mentions that “IQAir AirVisual platform AQI readings are based on the U.S. Environmental Protection Agency (EPA) National Ambient Air Quality Standards (NAAQS) to calculate AQI. Therefore, the use of the units “μg/m^3^” with the AQI value in the above-mentioned statements is incorrect and maybe wrongly interpreted as indicating a specific species.

2. Misconceptions of smog and its formation: The authors stated that (a) *The major reason for this deteriorated air quality in winter months is smog and (b) “The colder temperatures in initial months of winters lead to smog formation which blankets the major cities of Punjab (their reference 2)*.

The cited reference (their reference 2) has not discussed winter smog, instead, the authors considered crop residue burning, higher emissions of smoke aerosols, and meteorological conditions in November as the main source of winter smog, e.g., “For years, Pakistani environmentalists have referred to November, when crop burning, higher emissions, and cold weather combine to blanket Lahore and the rest of Punjab Province with acrid smog.” Smog is a mixture of smoke and fog, which normally occurs during mid/late autumn in Pakistan. Fog is formed by tiny water droplets in the lower atmosphere and smoke is emitted from combustion processes such as in bricks kiln and steel mills, burning of garbage, growing numbers of vehicles on the road, and especially, frequent and intense crop residue burning. The combination of these two (smoke and fog) is responsible for the formation of smog over the region, in particular during favorable meteorological conditions, such as low wind speed and low boundary layers capped by a strong inversion, reducing the transport of pollutants out of the region. The mentioned cities in the published article do not face smog issues during winter due to very little burning of crop residue. High levels of smog in winter are not supported by the paper, as the authors discuss only the AQI for autumn (November) (Figure 1), not for winter.

Pakistan is facing severe air quality issues throughout the year for a long time and none of the Pakistani cities meet the World Health Organization (WHO) recommended Air Quality Guidelines (AQG) and Pakistan's National Environmental Quality Standards (Pak-NEQS) [3]. Due to the lack of air quality monitoring stations, only a few studies have been conducted thus far that address the air pollution problems in Pakistan. Recently, Bilal et al. [3] conducted a comprehensive study on the air pollution scenario in Pakistan using multi-source data and characterized the pollution in 80 cities of Pakistan based on ultra-fine particulate matter (PM_1_), fine particulate matter (PM_2.5_), coarse particulate matter (PM_10_), nitrogen dioxide (NO_2_), sulfur dioxide (SO_2_), and aerosol optical depth (AOD). This kind of research is helpful for society, the research community, and policymakers. Therefore, our commentary should be considered and published as a supplementary explanation of the discussion in this short communication.

## Provenance and peer review

Not commissioned, externally peer reviewed.

## Sources of funding

There is no role of the sponsors.

## Ethical approval

No patient is involved.

## Consent

No consent is required.

## Author contribution

Muhammad Bilal: Conceptualization, Methodology, Software, Validation, Formal analysis, Investigation, Resources, Data curation, Writing—original draft Gerrit de Leeuw: Investigation, Writing—review and editing Janet E Nichol: Writing—review and editing Max P. Bleiweiss: Writing—review and editing Leiku Yang: Data curation Huabin Chua: Data curation Alaa Mhawish: Writing— review and editing Md. Arfan Ali: Data curation All authors have read and agreed to this version of the manuscript.

## Registration of research studies

1. Name of the registry: N/A
2. Unique Identifying number or registration ID: N/A
3. Hyperlink to your specific registration (must be publicly accessible and will be checked): N/A

## Guarantor

Muhammad Bilal.

## Declaration of competing interest

Nothing to disclose.
